# Gallbladder volvulus: it’s a small whirl

**DOI:** 10.1259/bjrcr.20150360

**Published:** 2016-05-23

**Authors:** Ben Layton, Velauthan Rudralingam, Rochelle Lamb

**Affiliations:** ^1^University Hospital South Manchester, Manchester, UK; ^2^North Manchester Hospitals, Manchester, UK

## Abstract

An 85-year-old female with an extensive past medical history attended our accident and emergency department with vague abdominal pain and distension. Clinical examination and initial blood tests demonstrated features of sepsis and she was commenced on broad spectrum antibiotics. Following admission, a contrast-enhanced CT scan of the abdomen and pelvis was performed, which showed an unusual configuration of the gallbladder. There was significant luminal distension of the gallbladder, which was found to lie in an unusual horizontal position anterior to the liver edge. This was a new finding compared with a recent CT pulmonary angiogram that demonstrated a normal gallbladder anatomy. In addition, there was abrupt angulation of the gallbladder neck, demonstrating a “beak” sign as well as indrawing of the vascular pedicle forming a mini “swirl” sign. The constellation of gallbladder distension, abnormal lie, “beak” and mini “swirl” sign together is a strong indicator of gallbladder torsion. Coexistent right lower lobe pneumonia was noted. The patient was too frail for surgical intervention and succumbed to underlying sepsis. Post-mortem examination demonstrated a gangrenous gallbladder secondary to volvulus. We present a rare case of gallbladder volvulus and highlight CT findings to help the radiologist make a pre-operative diagnosis.

## Summary

Only a few hundred cases of gallbladder volvulus have been published since the end of the 19th century, making it an uncommon diagnosis. It is reported that up to 10% of patients are diagnosed on imaging prior to surgery.^1,2^ There is a convincing preponderance for elderly females, and without prompt surgical attention, gallbladder volvulus is a life-threatening condition. Although a rare diagnosis, gallbladder torsion should be given some consideration in the elderly population with suspected cholecystitis refractory to conservative management. The first-line management differs from that for classical acute cholecystitis and the dilemma is that both conditions may present with only subtle differences. Imaging provides a means of diagnosing gallbladder volvulus early and there are specific signs on CT scans that may be present to raise suspicion of this diagnosis and potential vascular compromise. We present a case of gallbladder torsion in an elderly female with useful signs of this condition on CT scan.

## Clinical presentation and investigation

An 85-year-old female presented to the emergency department following a referral from her general practitioner with lethargy and abdominal pain. She had an extensive past medical history including diverticulosis, rheumatoid arthritis, depression, anxiety and alcohol dependence. On examination, she was found to have supraventricular tachycardia and abdominal distension. Her blood investigations demonstrated leukocytosis, acute kidney injury and an elevated C-reactive protein that were consistent with with underlying sepsis, and broad-spectrum i.v. antibiotics were commenced. She had an abdominal radiograph that showed no evidence of intestinal obstruction or perforation. She remained under the care of the medical team and a surgical opinion was sought for further investigation of her persistent abdominal distension.

**Figure 1.**
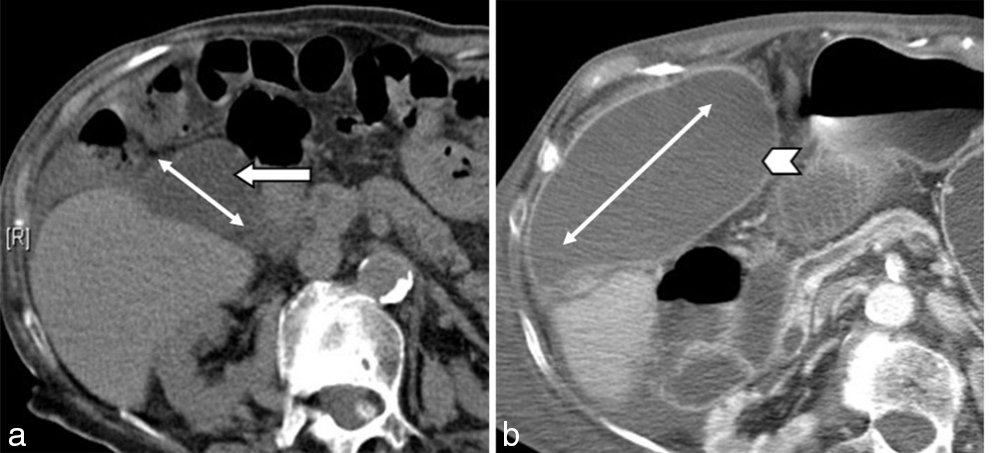
(a) Axial slice from a recent CT pulmonary angiogram used for comparison demonstrates a gallbladder normal in size and position (arrow). The axis of the gallbladder from neck to fundus is highlighted (double-sided white arrow). (b) There is a dramatic change in the anatomical position of the gallbladder (arrowhead) with a horizontal lie and grossly distended state—two signs of gallbladder volvulus. The striking change in the axis (double-ended white arrow) is significant.

Given the patient’s stable clinical condition and lack of peritoneal signs, an emergency CT scan was not undertaken until day 2 of her admission. The CT image showed no evidence of mechanical intestinal obstruction or features of bowel ischaemia. The gallbladder was noted to lie in an unusual horizontal axis anterior to the liver surface, mimicking a collection (Figure 1). At the time of the CT scan, the gallbladder wall demonstrated normal enhancement without any adjacent fat stranding or fluid. There was also coexistent lobar pneumonia. The patient continued to receive supportive care but failed to recover and following an acute deterioration, died on day 7 of admission.

## Outcome

**Figure 2.**
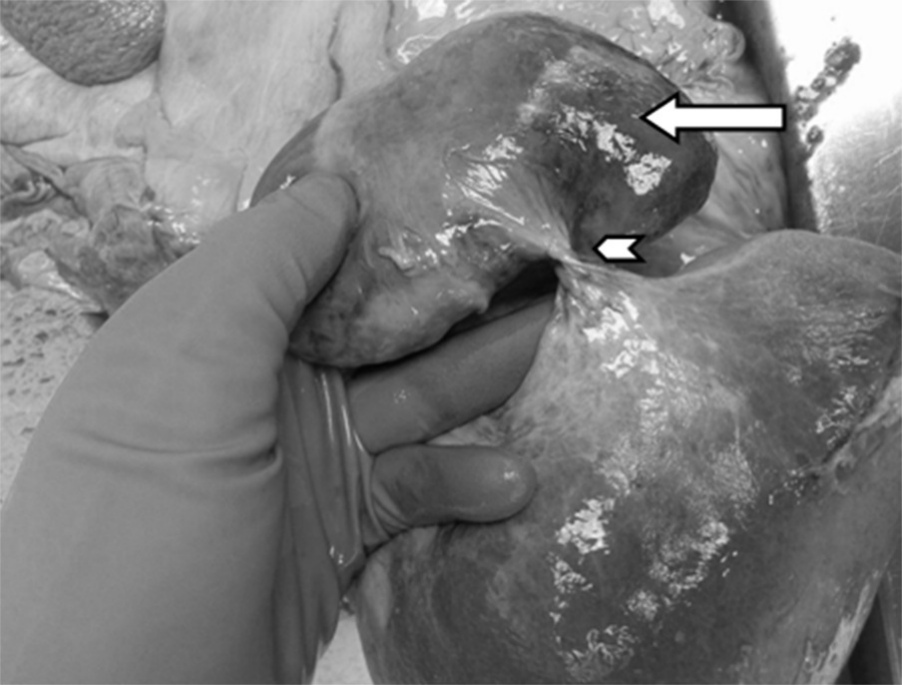
Photograph from the post-mortem examination showing a distended and necrotic gallbladder (arrow) twisted on a fulcrum (arrowhead). The twist involves the vascular pedicle.

A post-mortem examination revealed the cause of her death to be secondary to torsion of the gallbladder. The findings were of a distended necrotic gallbladder with a twist at the neck involving the vascular pedicle (Figure 2). The gallbladder wall had become gangrenous secondary to torsion, causing vascular compromise. The lumen of the gallbladder contained haemorrhagic exudative material.

## Discussion

**Figure 3.**
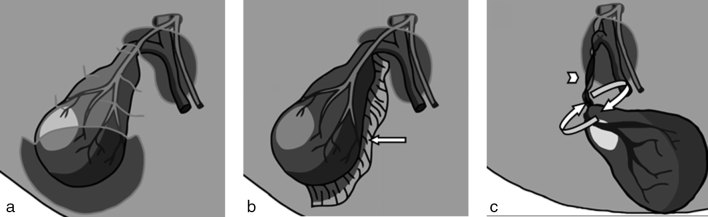
Sketches of the gallbladder viewed from behind showing two anatomical variants (a and b) and loss of adherence to the liver predisposing to torsion (c). (a) Normal anatomy of a gallbladder adherent to the liver covered by the peritoneum, cut away in this sketch. (b) Normal anatomical variant—gallbladder held to the liver by a short, broad mesentery (arrow). (c) Gallbladder torsion—the gallbladder body is not fixed to the liver and is suspended from a point, which is a risk factor for torsion. Note the twist (curved arrows) on the vascular pedicle (arrowhead), distension and abnormal horizontal lie.

Gallbladder volvulus is a potentially life-threatening condition. This is a rare condition and can easily be overlooked owing to the clinical presentation overlapping with that of classical acute cholecystitis. Since it was first recognized in the late 19th century, there have been only a few hundred documented cases. The literature describes an elderly female preponderance (3 : 1) aged between 60 and 80 years, although around 50 paediatric cases have now been recorded.^3^ The anatomy of the gallbladder and its relationship with the liver is variable.^4^ Its ability to twist on a point is limited if it is adherent to the liver and covered by the peritoneum. The gallbladder may also be held to the liver by a mesentery, which, if short and broad-based, also hinders torsion. However, if the mesentery is particularly long or attaches to the gallbladder only at the cystic duct, the gallbladder will lie suspended freely from a point within the peritoneal cavity and there will be a predisposition to twisting (Figure 3).^5^ This predisposition is not just a congenital one; atrophy of the liver and loss of elastic tissue with age and loss of supporting structures such as fat may also free the gallbladder, allowing it to twist on a pedicle.^2,6^ This configuration likely accounts for the aetiology in this case.

For the most part, the appearance of gallbladder volvulus on a CT scan is not pathognomonic of the condition. It can appear akin to classical cholecystitis with superimposed features of gangrene. Attempts have been made to outline the criteria on CT scans to aid in the diagnosis of gallbladder volvulus. Kitigawa et al^7^ described four diagnostic criteria: fluid collection in the gallbladder fossa, a shift in the axis of the gallbladder from vertical to horizontal, a hyperenhancing cystic duct to the right of the gallbladder and features of acute inflammation of the gallbladder wall.

We have found all but one of these criteria to be non-specific. Some of the signs described can be seen in severe acute cholecystitis alone. The crucial distinguishing feature is recognizing the “twist” along the vascular pedicle of the gallbladder. This may manifest as a shift in the axis of the gallbladder, typically from a normal vertical to a horizontal lie. In our patient, a recent CT pulmonary angiogram demonstrated a normal anatomical position of the gallbladder in the gallbladder fossa.

**Figure 4.**
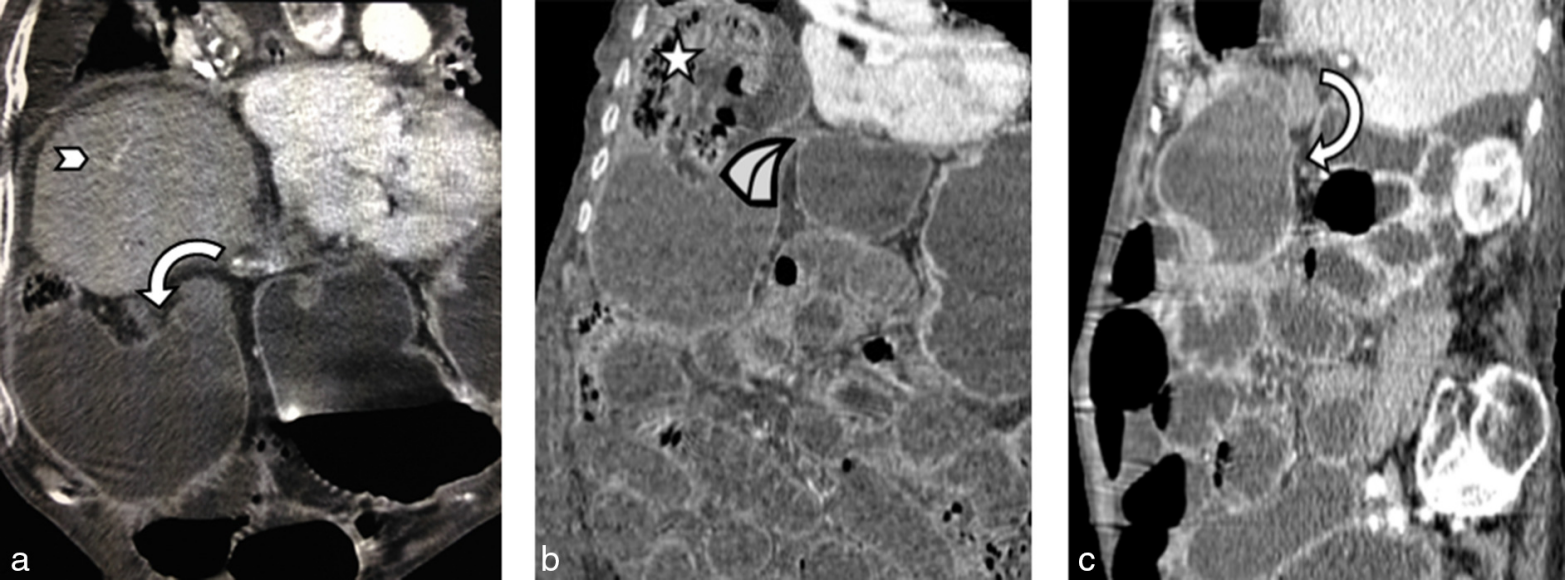
CT multiplanar reconstructions demonstrating the crucial beak and swirl features of gallbladder volvulus. (a) Coronal oblique reconstruction. There is gross distension of the gallbladder, which is in a horizontal lie. There is abrupt angulation at the Hartmann’s pouch (curved arrow). The arrowhead denotes the right lobe of the liver. (b) Coronal reconstruction. Angulation at the Hartmann’s pouch gives rise to a “beak” sign (highlighted with a sketch of a beak). Note the incidental Chilaiditi’s sign (star). (c) Sagittal image. Curved arrow demonstrates a mini “swirl” sign at the point of angulation, which represents a gallbladder volvulus (curved arrow).

At the point of the twist, there is a transition from the distended lumen to a fulcrum point that resembles a curved beak. Equally, the vascular pedicle and the surrounding fat dive in and form a mini “swirl” appearance. These two signs, more commonly associated with bowel volvulus, can also be seen in case of gallbladder volvulus. There is often coexistent strangulation of the gallbladder wall. In this case, at the time of the scan, the gallbladder wall demonstrated normal uniform enhancement, without any overt features of necrosis. By the time of the post-mortem examination, twisting of the vasculature had disrupted the blood supply to the gallbladder and led to gangrene. Together with the axial views (Figure 1), the use of multiplanar reformats greatly aids the detection of the above signs (Figure 4).

Hence the combination of the three signs listed below on CT scan is a strong indicator of the diagnosis of an underlying gallbladder volvulus:

1. gallbladder distension
2. the presence of a “beak and swirl sign” immediately distal to the fulcrum point
3. change in the anatomical position of the gallbladder from vertical to horizontal.

Approximately 70% of cases of gallbladder volvulus are seen in the absence of stones.^3^ This reflects the anatomic predisposition of this condition rather than the “obstruction to infection” sequence seen when gallstones cause conventional acute cholecystitis.

Coupling the constellation of CT findings with information about the patient adds weight to the radiologists’ pre-operative diagnosis. The literature shows the typical patient to be

- female
- aged 60–80 years
- scoliotic
- with tender, mobile right upper quadrant mass on examination—the “floating gallbladder” sign.^6,8^

Pre-operative diagnosis is challenging; however, while it was previously unheard of, there has been increased awareness of the condition and a recent review discovered improved numbers of pre-operative diagnosis.^3^ Awareness of CT scan findings, particularly the three specific signs described above, can help the increase diagnostic accuracy.

## Conclusions

Prospective diagnosis of gallbladder volvulus remains difficult; however, the growing bank of case reports around this rare diagnosis has provided a collection of very useful signs the radiologist can bear in mind where it is in the differential. This case demonstrates that gallbladder volvulus, if not diagnosed in a timely manner, is associated with high mortality and describes three specific features of the condition to aid the radiologists in their report.

## Learning points

1. Gallbladder volvulus is an uncommon diagnosis and associated with high mortality.
2. The diagnosis of gallbladder volvulus must be considered with the following CT scan findings: a distended gallbladder with change in axis, a “beak” and “swirl” appearance at the gallbladder neck and the absence of gallstones.
3. Once this diagnosis is considered, recommend prompt surgical exploration.

## Consent

Informed consent has been obtained and is held on record.
